# Supplementary material for: Identification of microbial antigens in liver tissues involved in the pathogenesis of primary biliary cholangitis using 16S rRNA metagenome analysis
Source: PLoS One. 2024 Aug 19;19(8):e0308912. doi: 10.1371/journal.pone.0308912 (PMC11332946; doi:10.1371/journal.pone.0308912)
Supplement: S1 Table — (DOCX) [file pone.0308912.s001.docx]

| Forward | 5’-GAGAGCGTCTATTCGATG-3’ |
| --- | --- |
| Reverse | 5’-CAGCGAGGTGGTGAAGATG-3’ |

The primers to detect both *S. panacis* and *S. paucimobilis* reference species
