# Supplementary figures and images for: Identification of microbial antigens in liver tissues involved in the pathogenesis of primary biliary cholangitis using 16S rRNA metagenome analysis
Source: PLoS One. 2024 Aug 19;19(8):e0308912. doi: 10.1371/journal.pone.0308912 (PMC11332946; doi:10.1371/journal.pone.0308912)

Figure 3.

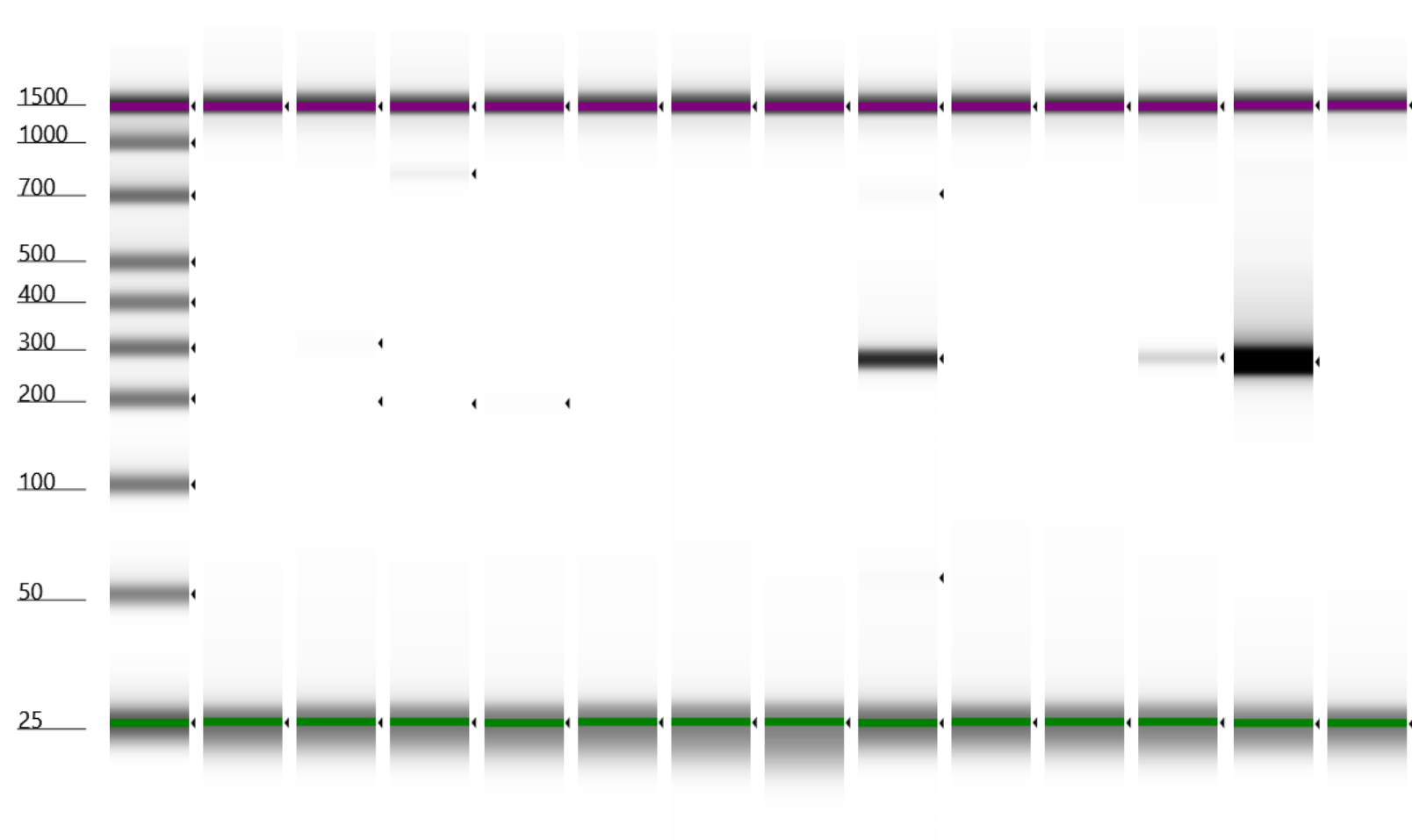

**Figure 4.**

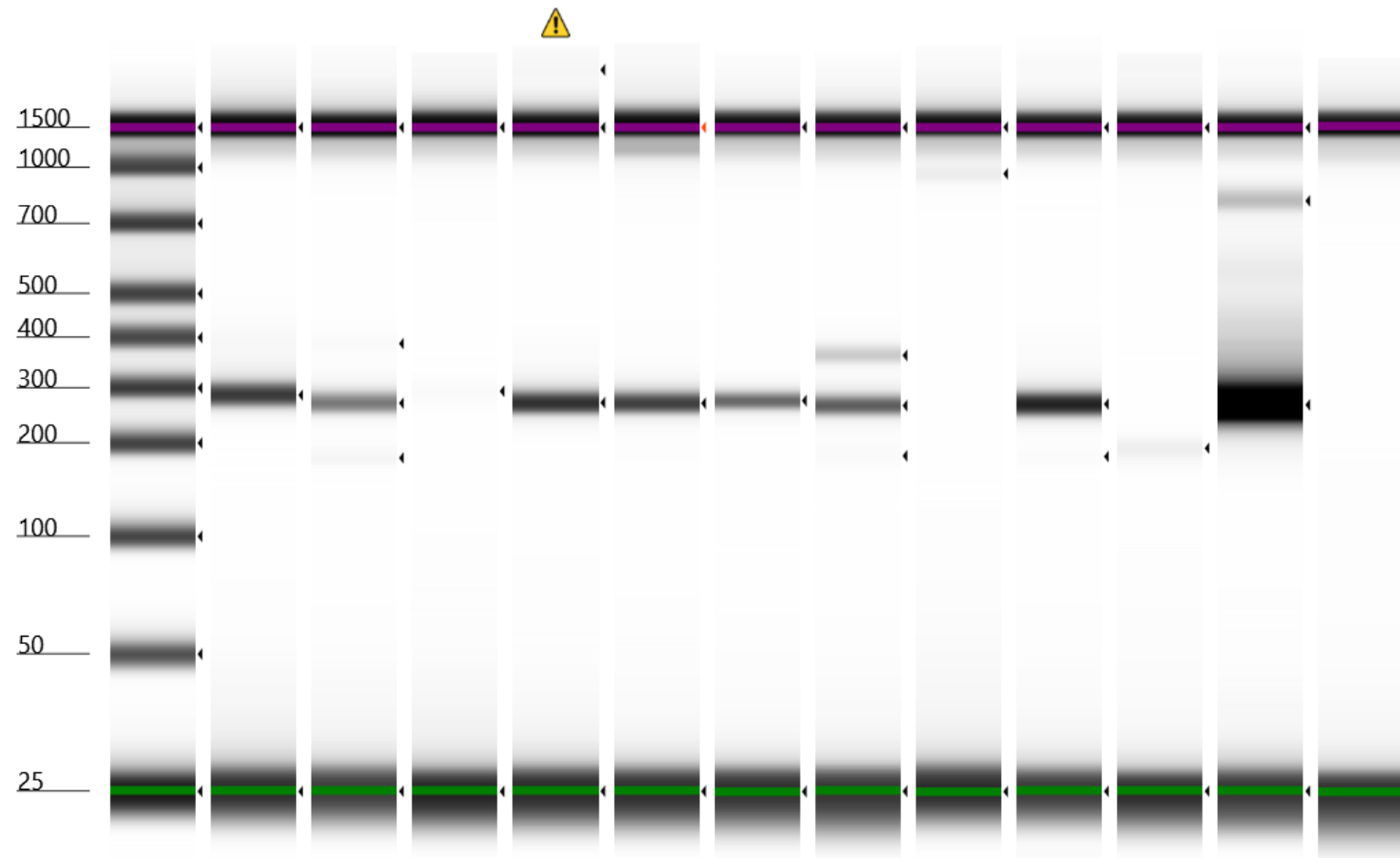

Supplement: S1 Raw images — (PDF) [file pone.0308912.s006.pdf]
